# Supplementary material for: Multiscale analysis and functional validation of the cellular and genetic determinants of skeletal disease
Source: bioRxiv. 2026 Jun 1:2024.12.16.628792. Preprint. [Version 2] doi: 10.1101/2024.12.16.628792 (PMC13251937; doi:10.1101/2024.12.16.628792)

Extended Data Fig. 1. Genes and gene programs that define chondrocytes, endothelial cells, vascular smooth muscle cells and osteoclasts

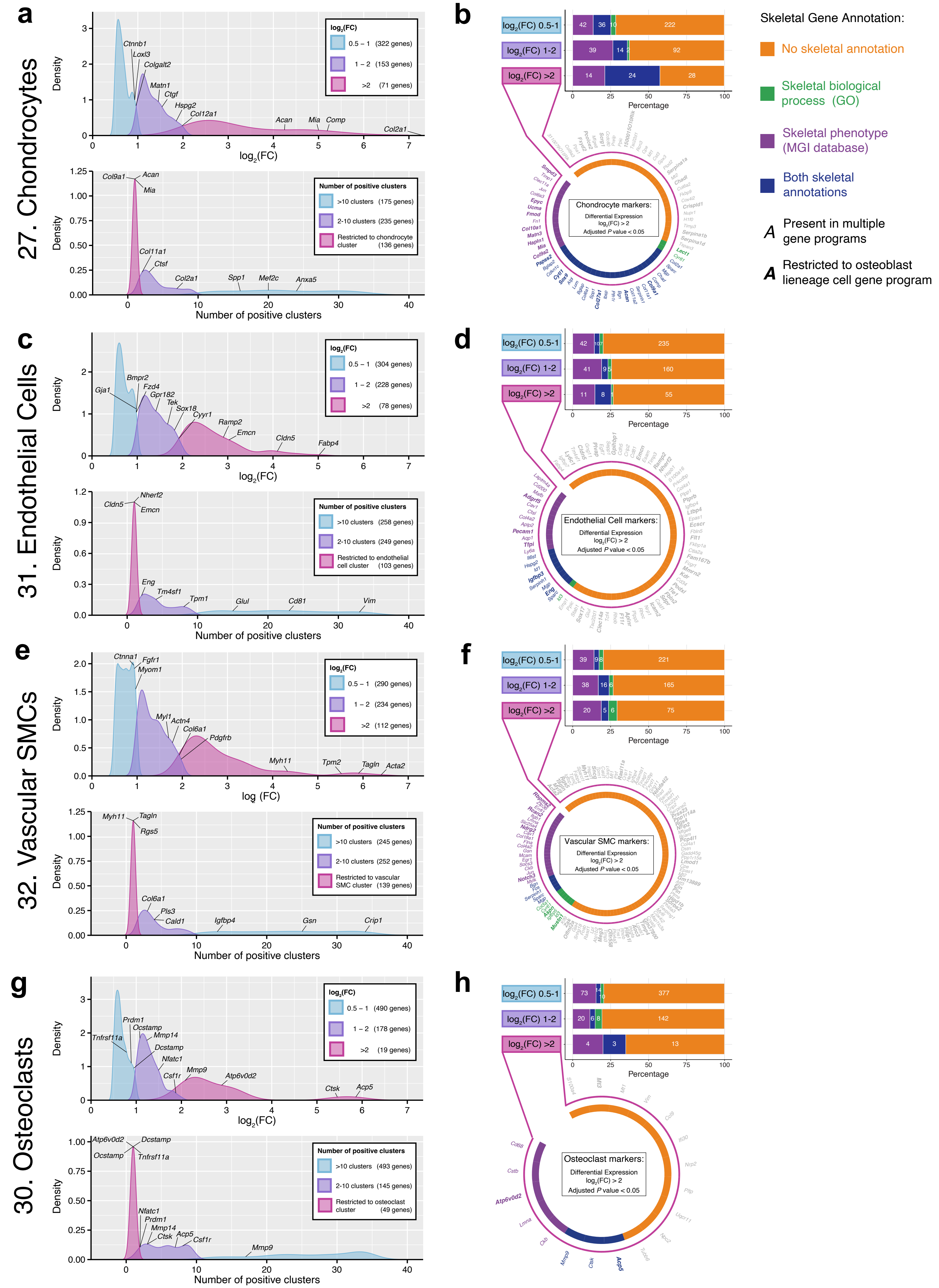

Supplement: Supplement 1 — Analysis of gene programs that define the chondrocyte (cluster 27), endothelial cell (cluster 31), VSMC (cluster 32) and osteoclast (cluster 30) clusters from Fig. 1c. (a) Density plots of log2fold-change [log2(FC)] in expression of genes (top) and total number of clusters expressing genes (bottom) that define the chondrocyte gene program (cluster 27). (b) Waffle plots for individual genes (coloured squares) in the chondrocyte gene program with log2(FC) 0.5–1 (top), log2(FC) 1–2 (middle) and log2(FC) >2 (bottom) in expression. Genes annotated with a skeletal process in the GO database (green), MGI database (purple), in both databases (blue) or are unannotated (orange) are shown. Circos plot shows individual genes with a log2(FC) >2, with those found only in the chondrocyte cluster gene program indicated in bold. (c) Density plots of log2(FC) in expression of genes (top) and total number of clusters expressing genes (bottom) that define the endothelial cell gene program (cluster 31). (d) Waffle plots and circos plot for individual genes in the endothelial gene program. Annotated as described in (b). (e) Density plots of log2(FC) in expression of genes (top) and total number of clusters expressing genes (bottom) that define the VSMC gene program (cluster 32). (f) Waffle plots and circos plot for individual genes in the VSMC gene program. Annotated as described in (b). (g) Density plots of log2(FC) in expression of genes (top) and total number of clusters expressing genes (bottom) that define the osteoclast gene program (cluster 30). (h) Waffle plots and circos plot for individual genes in the osteoclast gene program. Annotated as described in (b). [file media-1.pdf]
