# Supplementary figures and images for: Multiscale analysis and functional validation of the cellular and genetic determinants of skeletal disease
Source: bioRxiv. 2026 Jun 1:2024.12.16.628792. Preprint. [Version 2] doi: 10.1101/2024.12.16.628792 (PMC13251937; doi:10.1101/2024.12.16.628792)

**a**

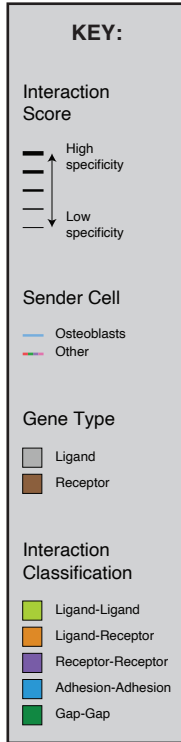

## b

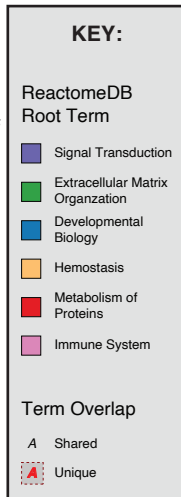

Supplement: Supplement 2 — (a) Circos plots displaying predicted interactions between osteoblast lineage cells and selected cell types. Genes are arranged according to the annotated pathways present within CellPhoneDB and labelled accordingly in the outer layer. Interaction types are indicated by coloured boxes. Line colour indicates which cell type is the “sender” cell type for a given interaction, whilst thickness reflects interaction specificity. For clarity, only interactions with a specificity score ≥50% are included within the plots. (b) Bar plots displaying enriched ReactomeDB pathways identified amongst interactions between osteoblast lineage cells and selected cell types. Pathways are grouped according to their root term within the ReactomeDB database (see Methods). Adjoining numbers indicate the proportion of genes associated with a term that were present within the results. Terms that are uniquely enriched for one cell type are indicated in grey boxes and red outlines/text. [file media-2.pdf]

# Extended Data Fig. 3. High-resolution clustering of non-haematopoietic cells

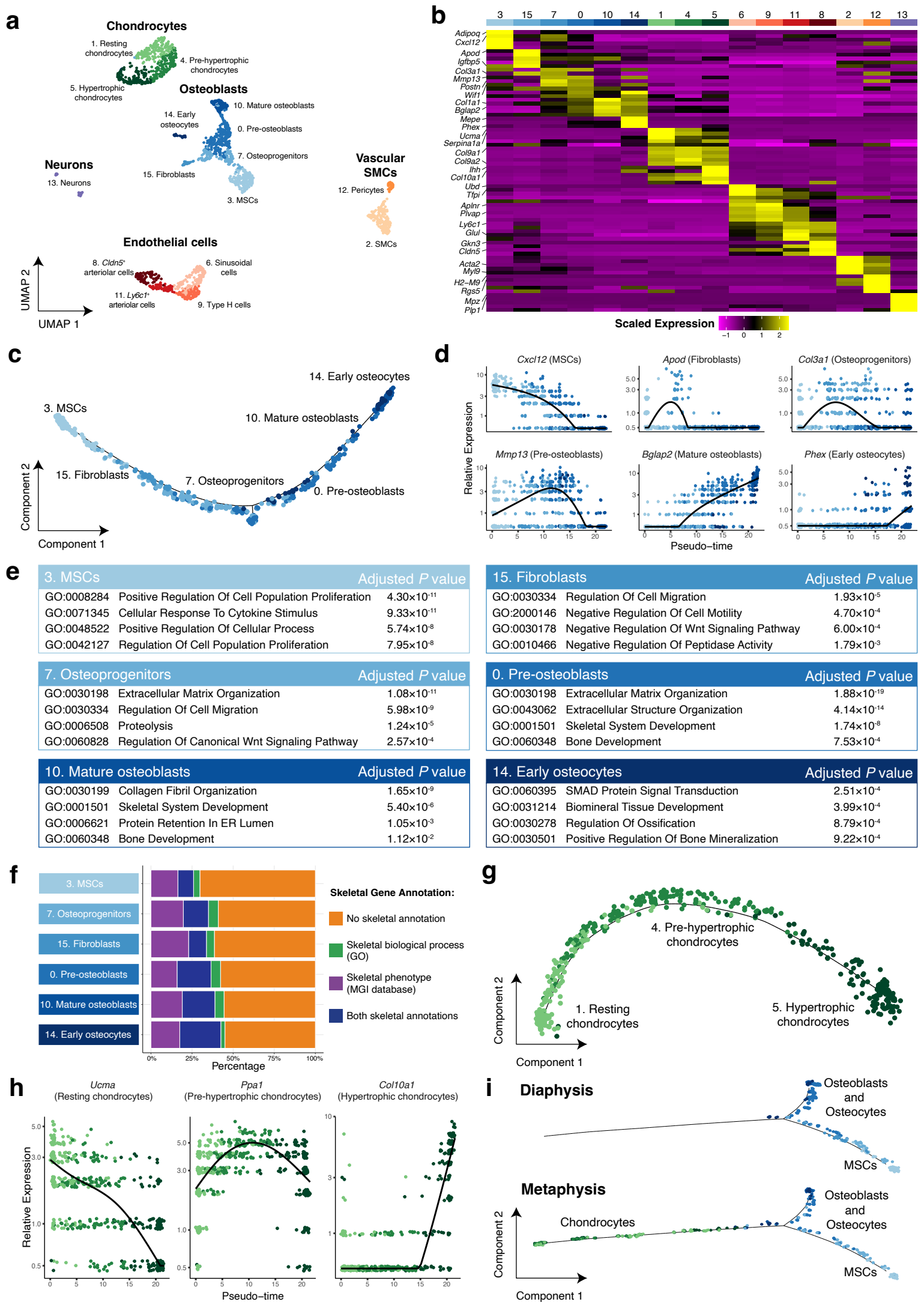

Supplement: Supplement 3 — (a) UMAP of cell clusters identified in osteoblast lineage, chondrocyte, endothelial cell, VSMC and neuronal cell types. (b) Heatmap of the top 10 genes expressed by each of the non-haematopoietic sub-clusters ranked by log2(FC). Two example genes that define each sub-cluster are annotated. (c) Predicted relationship between individual osteoblast sub-clusters determined by pseudo-time trajectory analysis. (d) Expression of exemplar genes of each osteoblast sub-cluster in pseudo-time. (e) Gene ontology analysis of the gene programs for each osteoblast sub-cluster. (f) Bar plot showing the proportion of genes in osteoblast sub-cluster gene programs. Genes annotated with a skeletal process in the GO database (green), MGI database (purple), in both databases (blue) or are unannotated (orange) are shown. (g) Predicted relationship between individual chondrocyte sub-clusters determined by pseudo-time trajectory analysis. (h) Expression of exemplar genes of each chondrocyte sub-cluster in pseudo-time. (i) Predicted relationship between individual osteoblast and chondrocyte sub-clusters in diaphysis and metaphysis determined by pseudo-time trajectory analysis. [file media-3.pdf]

Extended Data Fig. 4. Analysis of transcription factor activity in non-haematopoietic cells

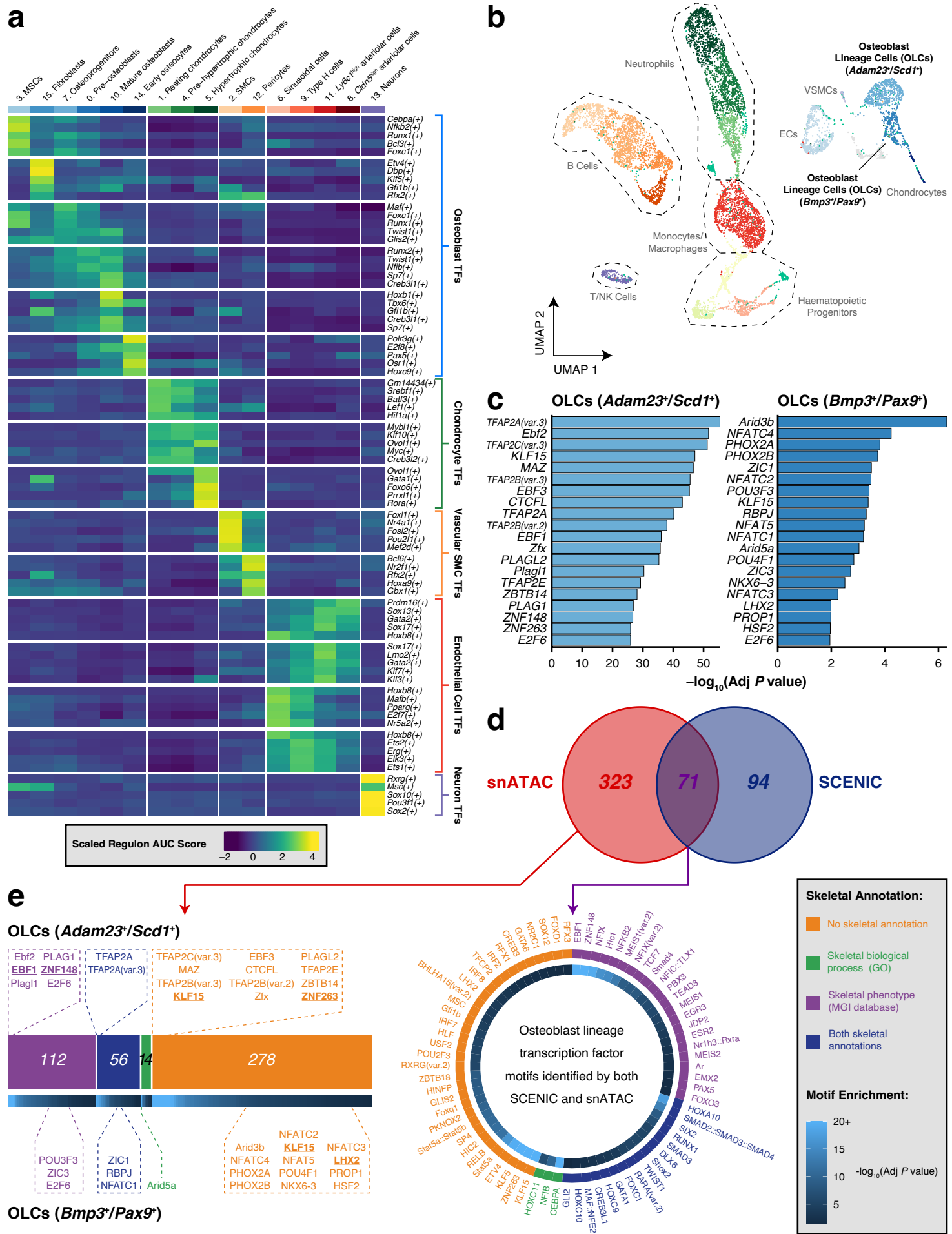

Supplement: Supplement 4 — (a) Heatmap displaying the mean activity of the target genes (regulons) for the top 5 transcription factors predicted by SCENIC to regulate each non-haematopoietic sub-cluster. Scale bar represents AUCell scores which indicate relative expression levels of the regulon across different cell types. (b) UMAP of cell clusters identified by snATAC-seq. (c) Barplots displaying the top enriched transcription factor binding motifs within differentially accessible peaks identified within the 2 osteoblast lineage cell (OLC) clusters. (d) Venn diagram showing overlap of identified transcription factors from SCENIC and snATAC-seq analyses. (e) Barplot displaying skeletal annotations of all transcription factors identified from snATAC-seq analysis. Top transcription factors for both osteoblast lineage clusters identified from snATAC-seq analysis in (c) are annotated. Common transcription factors identified from both snATAC-seq and SCENIC analyses are highlighted in bold and underlined. Circos plot displaying known skeletal annotations of all common transcription factors identified from both methods. Motifs involving genes annotated with a skeletal process in the gene ontology (GO) database (green), the mouse genome informatics (MGI) database (purple), in both databases (blue) or are not annotated in either (orange) are shown. Motifs are ordered by strength of enrichment with snATAC-seq analysis, as indicated by the lower bar in barplot and inner circle of circus plot. [file media-4.pdf]

**Extended Data Fig. 6. Genome-wide association study of eBMD in the UK Biobank study**

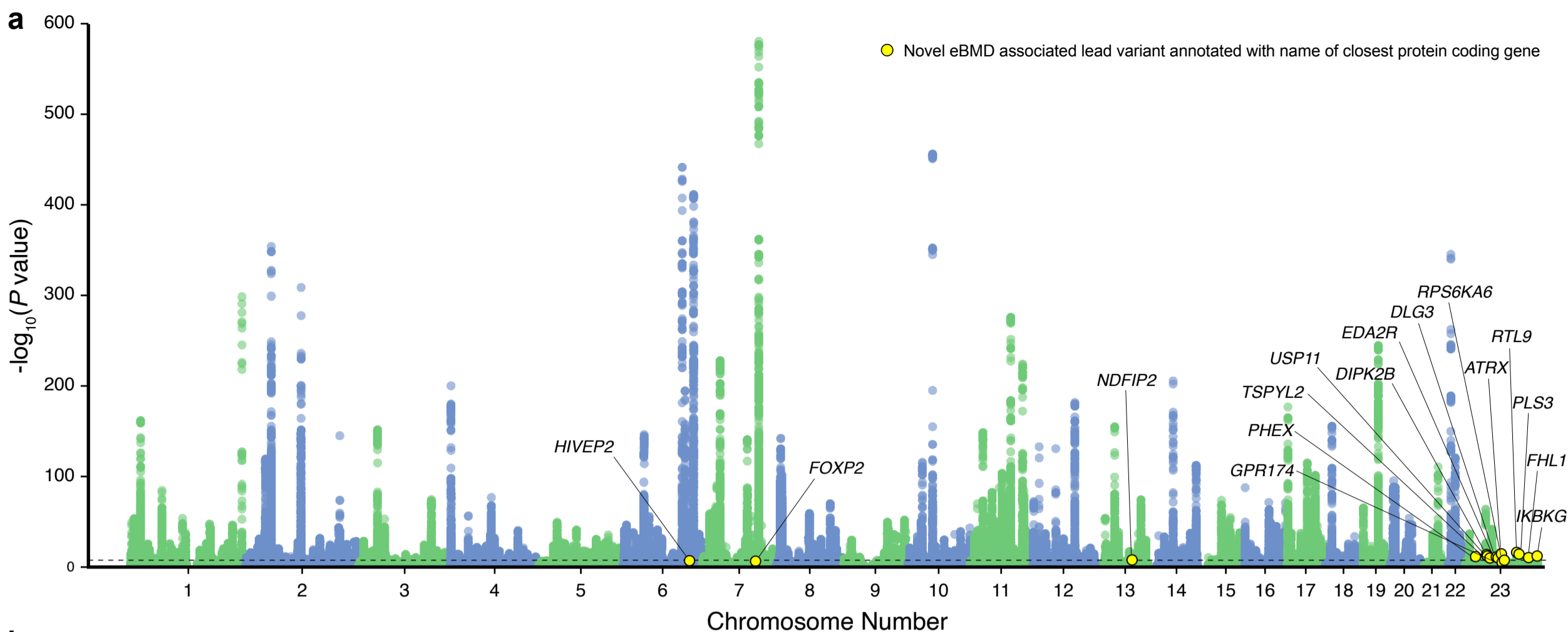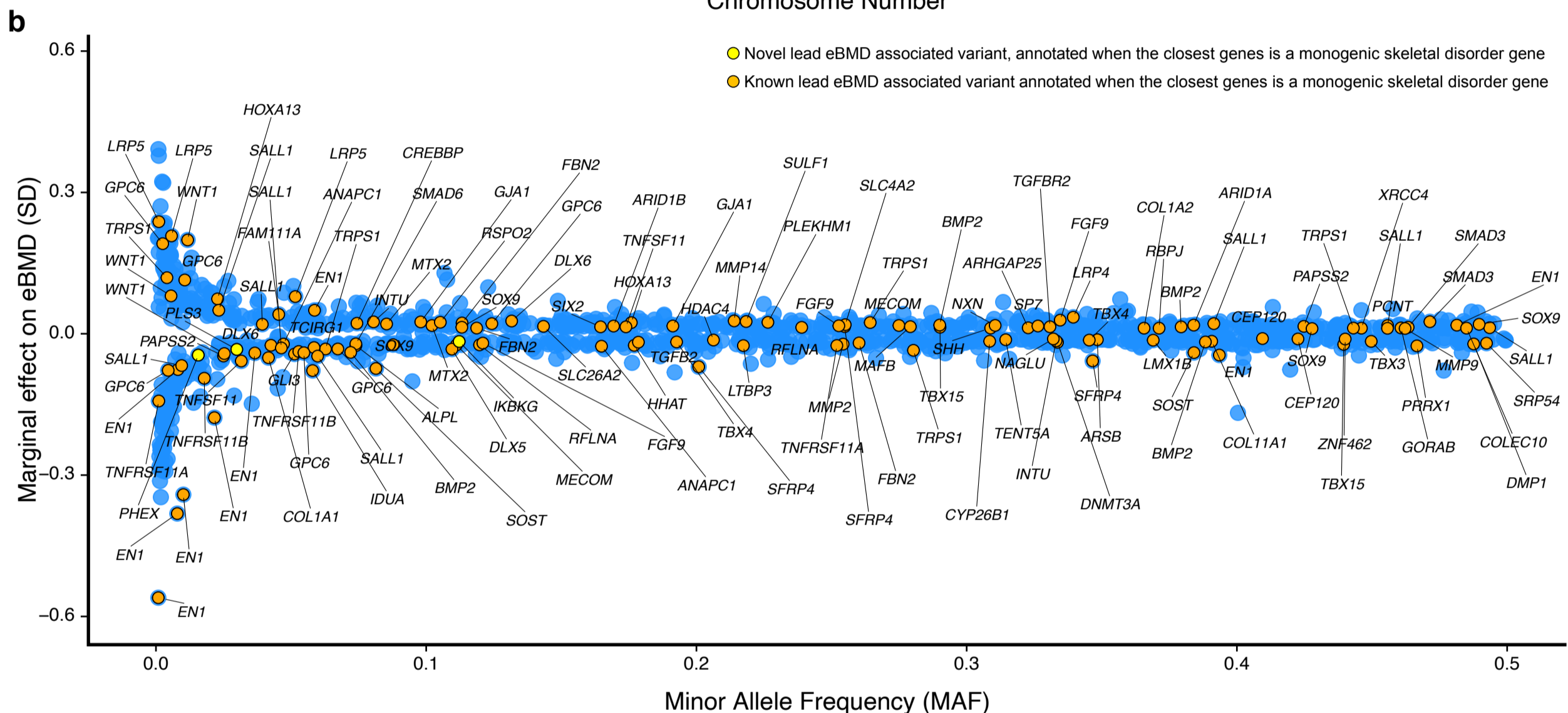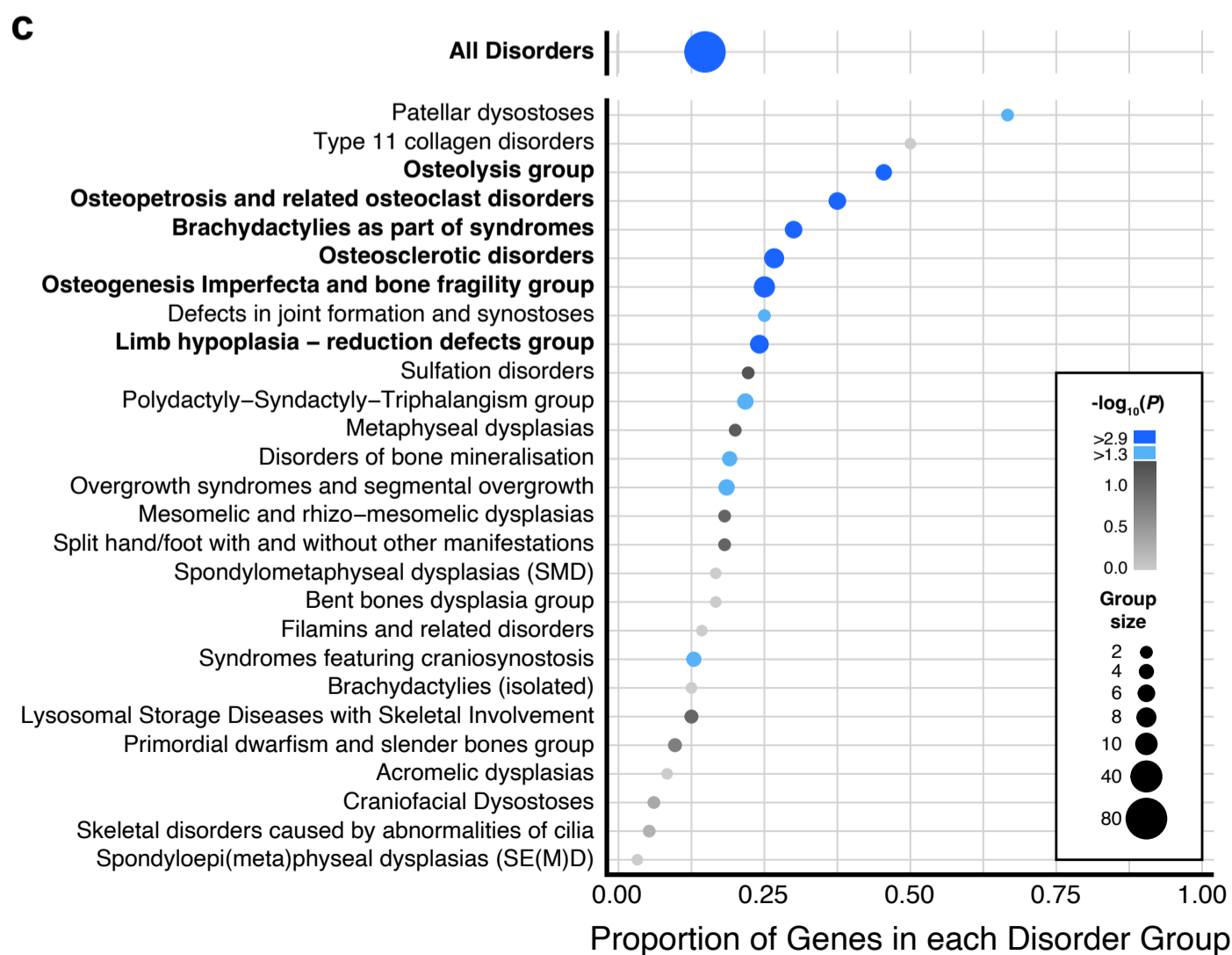

Supplement: Supplement 6 — (a) Manhattan plot summarising the results of eBMD GWAS performed on 448,010 participants in the UK Biobank study. The dashed line represents the threshold to declare genome wide significance (P < 6.6 × 10^−9^). Novel lead eBMD associated variants are coloured yellow and annotated with the name of the protein coding gene. The y axis is capped at −log10(P-value) = 600 and excludes 81 variants located within the WNT16/CPED1 locus on chromosome 7. Variants with P > 5.5 × 10^−5^ have also been excluded. (b) Bivariate scatter plot contrasting the magnitude of the genetic effect on eBMD and minor allele frequency of lead eBMD associated variants. Orange circles correspond to lead variants that map to known BMD associated loci, and yellow circles correspond to lead variants that map to novel loci. Gene symbols of monogenic skeletal disorder genes are annotated if they are located closest a lead eBMD associated variant. (c) Bubble plot showing the number, and proportion of monogenic skeletal disorder genes from each disorder group that are present in the set of 901 protein coding genes located closest to lead eBMD associated variants. Size of the circles represent the number of genes in each disorder group present within the gene list. Scale bar indicates the P value of enrichment, as determined by hypergeometric over-representation testing. Light blue dots indicate nominal evidence of enrichment: P value of <0.05 [−log10(P value) of >1.3]. Dark blue dots denote robust evidence of enrichment with Bonferroni-corrected threshold of 1.2 × 10^−3^ [−log10(P value) of >2.9]. [file media-6.pdf]

Extended Data Fig. 9. Cellular mechanism of skeletal phenotype in *Pls3* deficient mice

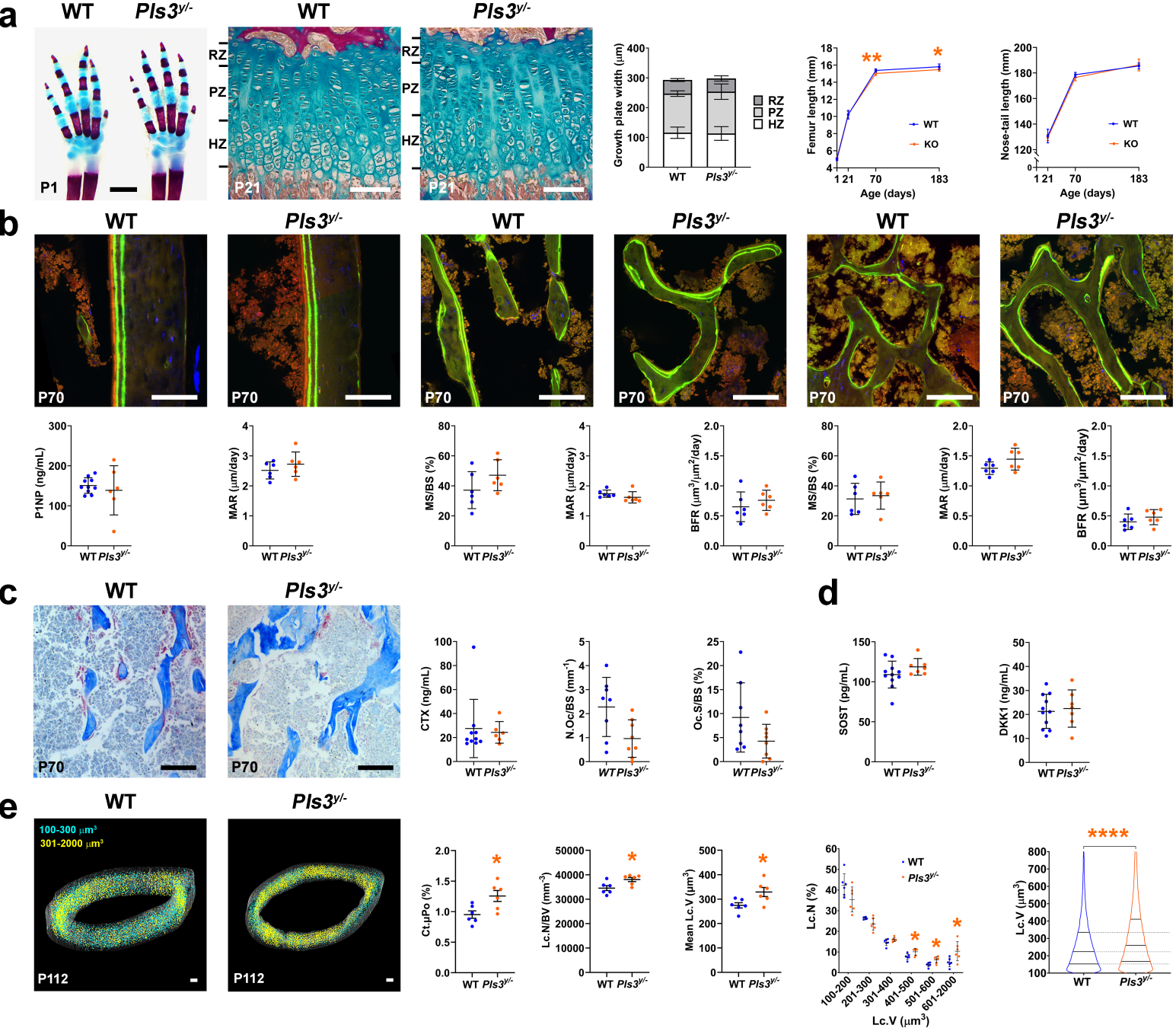

Supplement: Supplement 9 — (a) Left panel shows representative upper limb paws from P1 WT and Pls3y/− mice stained with alcian blue (cartilage) and alizarin red (bone); scale bar = 1mm. Second panel shows decalcified proximal tibia growth plate sections from P21 (WT n=5; Pls3y/− n=4) mice stained with alcian blue (cartilage) and van Gieson (bone osteoid); scale bar = 100μm. Left graph shows the absolute widths of the resting zone (RZ), proliferating zone (PZ) and hypertrophic zone (HZ) chondrocytes in the growth plate. Graphs show femur length from (P1 (WT n=6; Pls3y/− n=4), P21 (WT n=17; Pls3y/− n=8), P70 (WT n=10; Pls3y/− n=6), and P183 (WT n=12; Pls3y/− n=7) and nose to tail length from P21 (WT n=19; Pls3y/− n=8), P70 (WT n=8; Pls3y/− n=6), and P183 (WT n=12; Pls3y/− n=7); mean±SD;. Students’ t-test; *P<0.05; **P<0.01. (b) Confocal images of mid femur cortical bone, distal femur trabecular bone, and lumbar vertebral trabecular bone (L1-L3) from P70 (WT n=6; Pls3y/− n=6) mice, double-labelled with calcein; scale bar=100μm. Graphs show serum procollagen type 1 N propeptide (P1NP) levels P70 (WT n=10; Pls3y/− n=6) mice, femur cortical bone mineral apposition rate (MAR), femur trabecular bone mineralising surface per bone surface (MS/BS), MAR and bone formation rate (BFR), and lumbar vertebral trabecular bone MS/BS, MAR and BFR in P70 (WT n=6; Pls3y/− n=6) mice; mean±SD. (c) Decalcified sections of proximal tibia stained for tartrate-resistant acid phosphatase (TRAP) (osteoclasts) and aniline blue (bone) from P70 from (WT n=10; Pls3y/− n=6) mice; scale bar=100μm. Graphs show serum C-terminal telopeptide of type 1 collagen (CTX) levels P70 (WT n=10; Pls3y/− n=6) mice, numbers of osteoclasts per mm bone surface (OcN/BS), and osteoclast surface per mm bone surface (OcS/BS) in P70 (WT n=8; Pls3y/− n=8) mice; mean±SD. (d) Graphs show serum sclerostin (SOST) and dickkopf-related protein 1 (DKK1) levels in P183 (WT n=11; Pls3y/− n=7) mice. (e) Micro-CT images of a 250μm mid-femur ROI from P112 (WT n= [file media-9.pdf]

Extended Data Fig. 10. Spatial transcriptomics of human bone samples

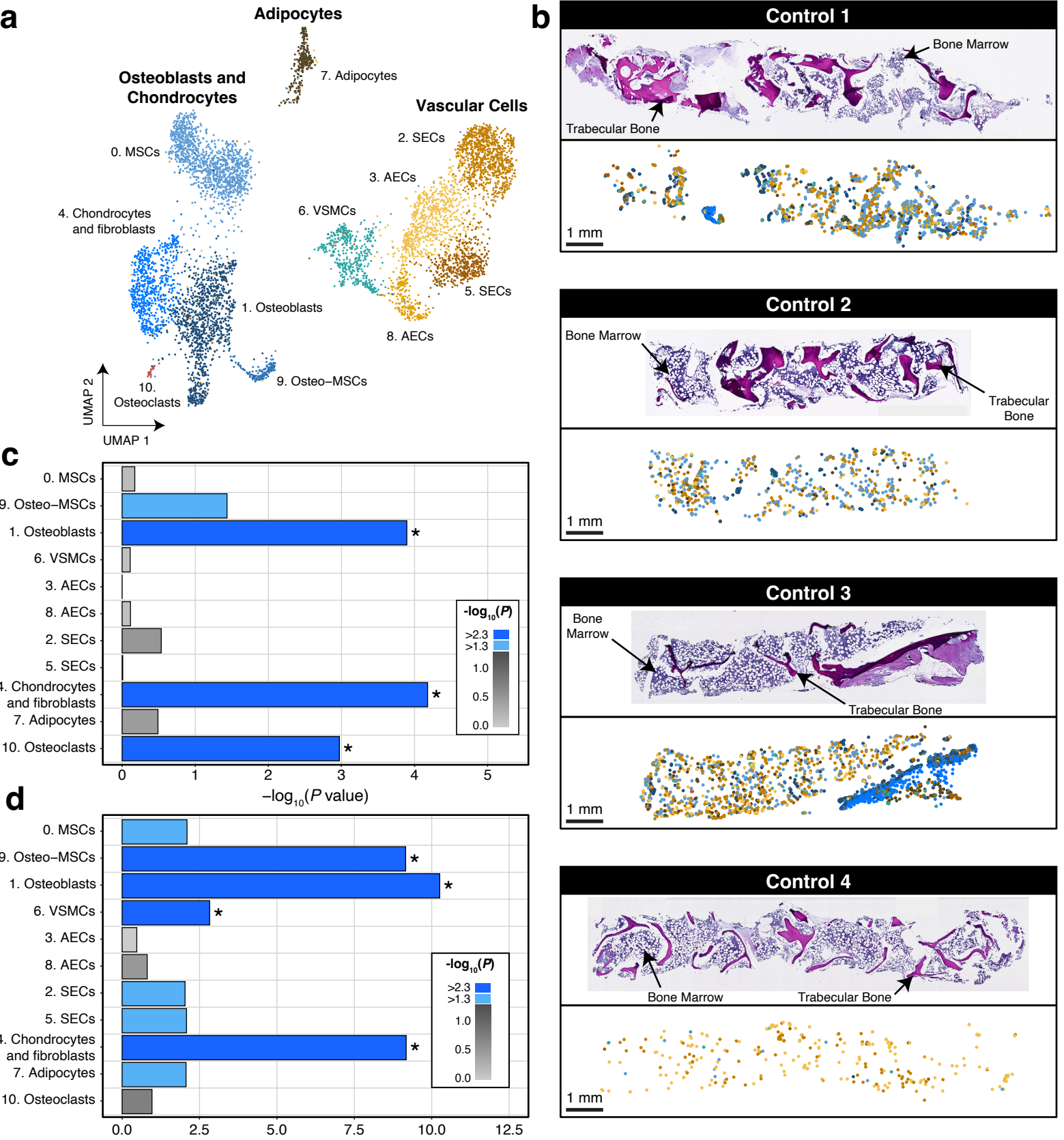

Supplement: Supplement 10 — (a) UMAP plot and of non-haematopoietic cell types and osteoclasts identified in trephine biopsies of 4 control patients as identified by Yip and Er et al52. MSCs = mesenchymal stromal cells; VSMCs = vascular smooth muscle cells; SECs = sinusoidal endothelial cells; AECs = arterial endothelial cells. (b) Haematoxylin and eosin (H&E) images and spatial plots of individual samples used for spatial transcriptomics. Spatial plots display only non-haematopoietic cells and osteoclasts for clarity and are coloured according to (a). Bar plots showing enrichment of gene programs for (c) causative genes of monogenic skeletal disorders and (d) eBMD-associated genes. Scale bars in bar plots indicate the P value. Light blue bars in bar plot correspond to observations that have nominal evidence of enrichment: P value of <0.05 [−log10(P value) of >1.3]. Dark blue bars and asterisks in bar plot correspond to observations that have robust evidence of enrichment and meet the Bonferroni-corrected significance threshold: P value of < 1.6 ×10^−3^ [−log10(P value) > 2.3]. [file media-10.pdf]

# Supplementary Fig. 5. Expression of exemplar genes in tissues outside of the skeleton.

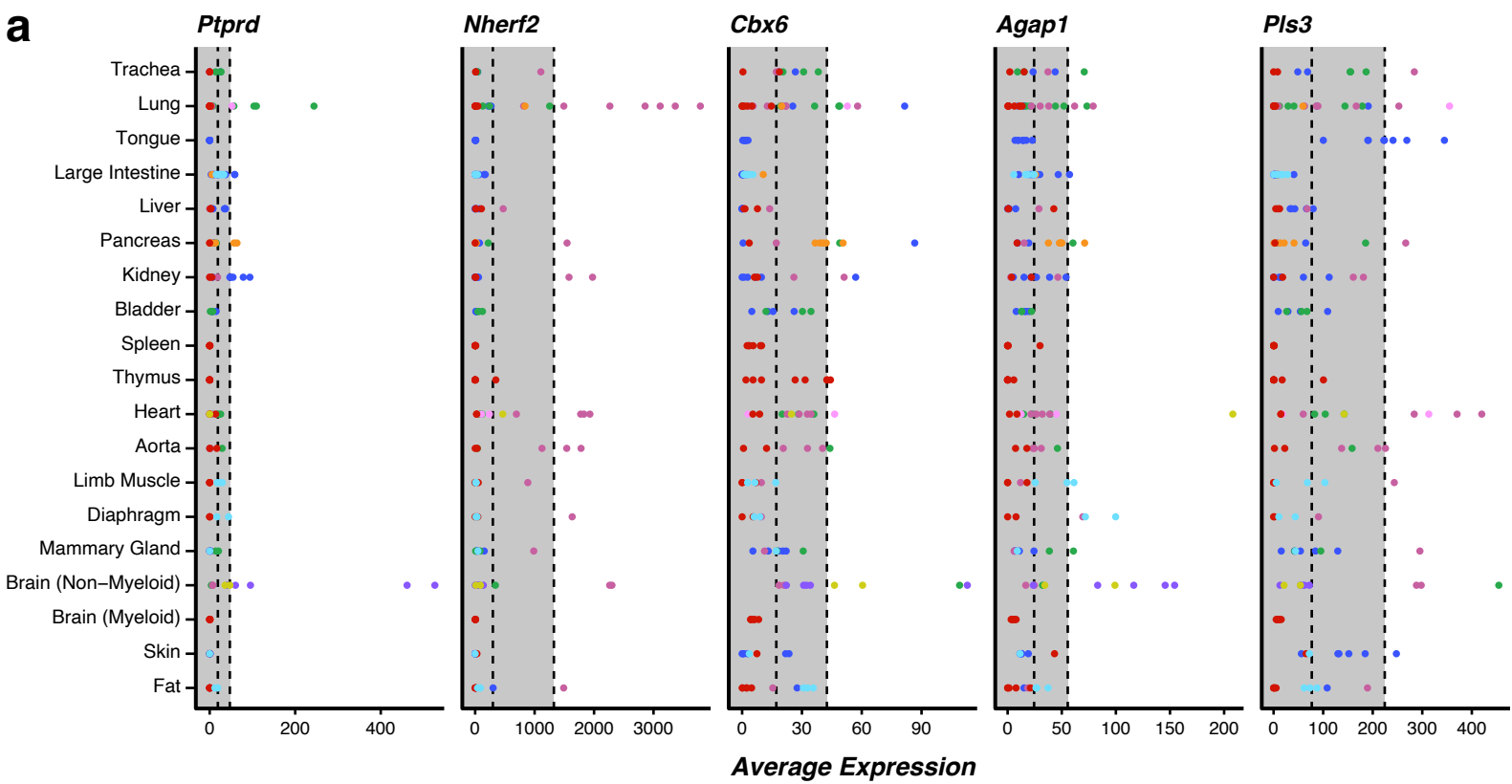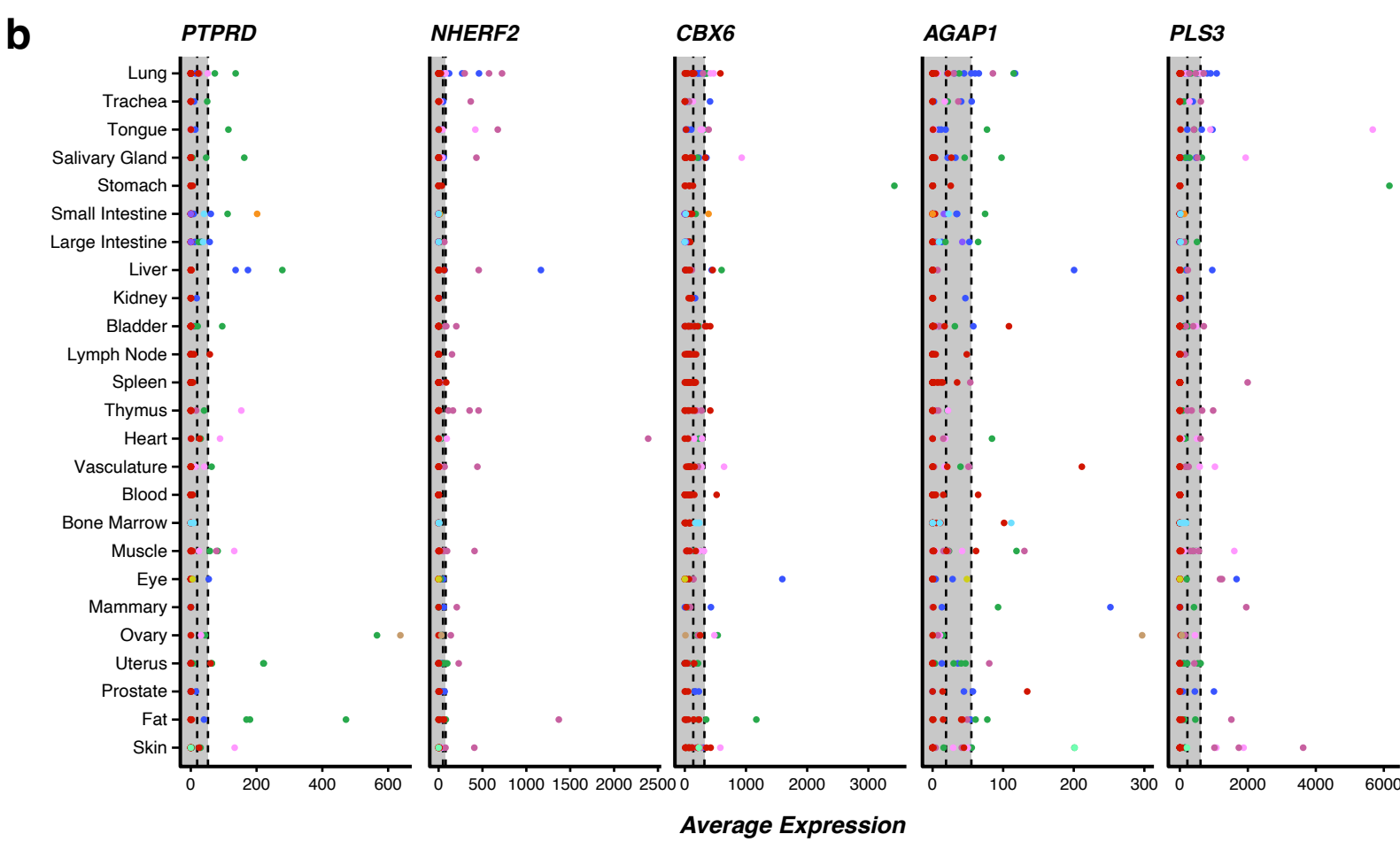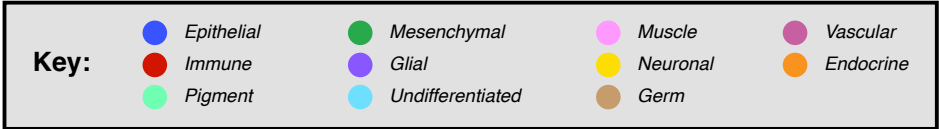

Supplement: Supplement 15 — Dotplots showing expression of exemplar genes across cell types isolated from different mouse (a) and human (b) tissues, using the TabulaMuris88 and TabulaSapiens89 datasets respectively. Individual cell types were manually assigned a classification using the Human Protein Atlas91 and coloured accordingly. Left dotted lines indicate the mean expression value of a gene across the whole dataset; right dotted lines indicate the 90th percentile value. [file media-15.pdf]

**Supplementary Fig. 6. Skeletal phenotyping of younger male *Pls3*<sup>y/-</sup> mice**

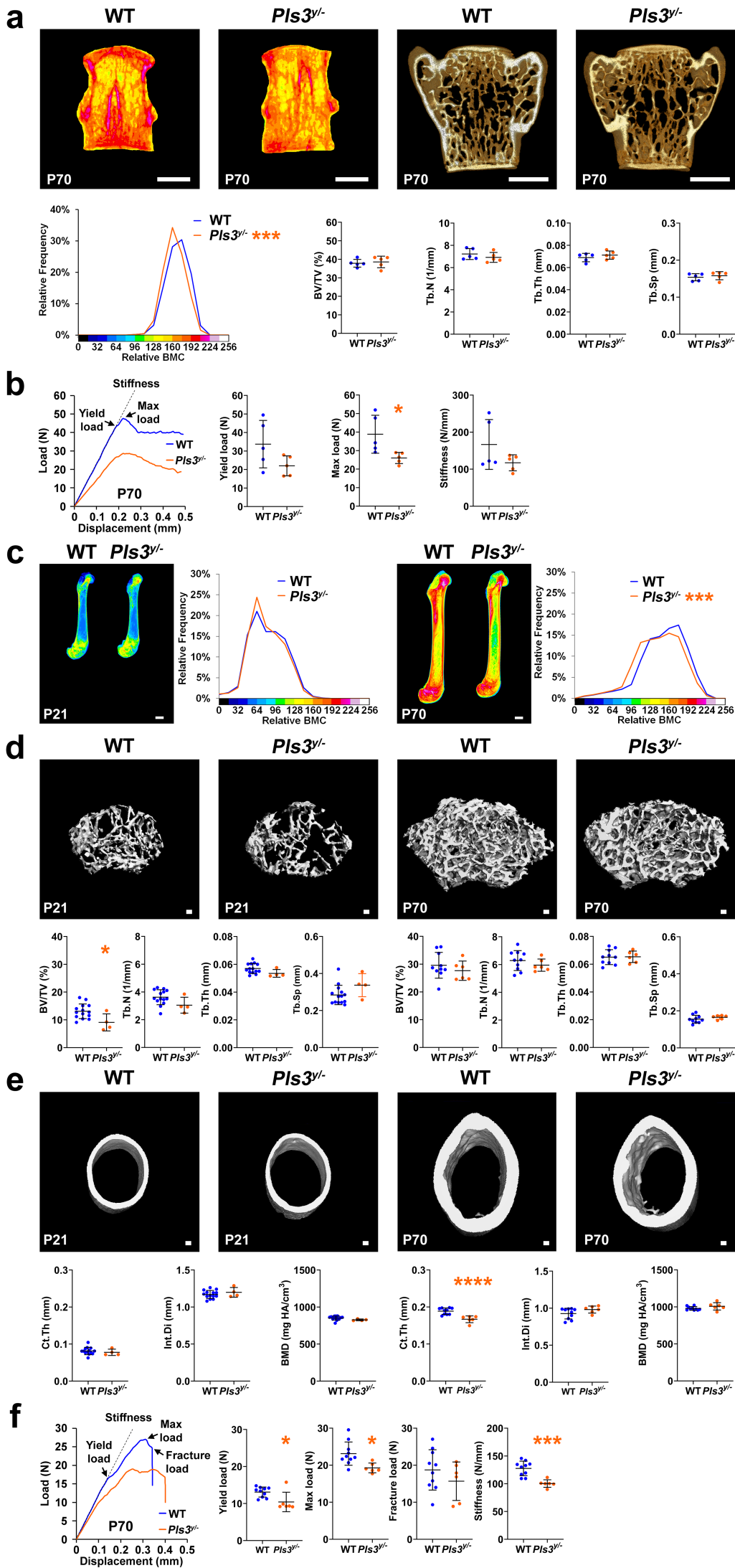

Supplement: Supplement 16 — (a) Pseudocoloured X-ray microradiography of lumbar vertebrae (L5) from P70 (WT n=5; Pls3y/− n=5) mice; scale bar = 1mm. Relative frequency histograms of BMC are shown for each age comparison; Kolmogorov-Smirnov test, ***P<0.001. Micro-CT images of mid coronal sections of lumbar vertebrae (L5) from P70 (WT n=5; Pls3y/− n=5) mice; scale bar = 1mm. Graphs show bone volume as a proportion of tissue volume (BV/TV), trabecular number (Tb.N), trabecular thickness (Tb.Th), and trabecular separation (Tb.Sp); mean±SD; Students’ t-test. (b) Representative load displacement curves from compression testing of lumbar vertebrae (L5) from P70 (WT n=5; Pls3y/− n=5) mice. Graphs show yield load, maximum load, and stiffness; mean±SD; Students’ t-test; *P<0.05. (c) Pseudocoloured X-ray microradiography of femurs from P21 (WT, blue, n=12; Pls3y/−, orange, n=4), and P70 (WT n=7; Pls3y/− n=6). Low bone mineral content (BMC) is blue/green and high BMC is pink; scale bar = 1mm. Relative frequency histograms of BMC are shown for each age comparison; Kolmogorov-Smirnov test, ***P<0.001. (d) Micro-CT images of distal femur trabecular bone from P21 (WT n=14; Pls3y/− n=4), and P70 (WT n=10; Pls3y/− n=6) mice; scale bar = 100μm. Graphs show bone volume as a proportion of tissue volume (BV/TV), trabecular number (Tb.N), trabecular thickness (Tb.Th), and trabecular separation (Tb.Sp); mean±SD; Students’ t-test; *P<0.05. (e) Micro-CT images of femur mid-shaft cortical bone from P21 (WT n=14; Pls3y/− n=4), P70 (WT n=10; Pls3y/− n=6) mice; scale bar = 100μm. Graphs show cortical thickness (Ct.Th), internal diameter (Int.Di), and bone mineral density (Ct.BMD); mean±SD; Students’ t-test; ****P<0.0001. (f) Representative load displacement curves from three-point bend testing of femurs from P70 (WT, blue, n=10; Pls3y/−, orange, n=6) mice. Graphs show yield load, maximum load, fracture load, and stiffness; mean±SD; Students’ t-test; *P<0.05, ***P<0.001. [file media-16.pdf]

Supplementary Fig. 7. Skeletal phenotyping of female *Pls3*<sup>y/-</sup> mice

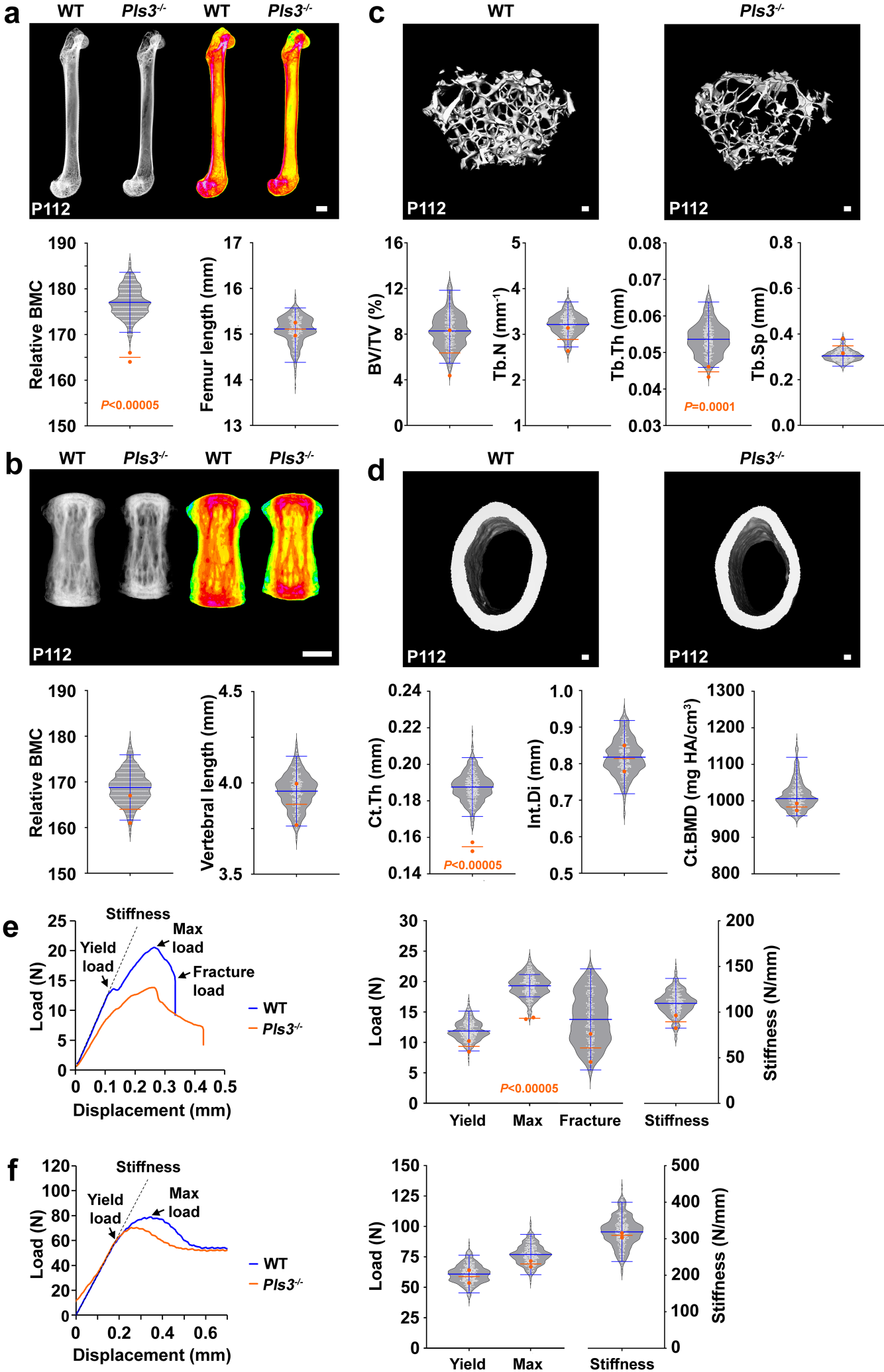

Supplement: Supplement 17 — (a) Greyscale and pseudocoloured X-ray microradiography of femurs from P112 WT and Pls3−/−mice. Low bone mineral content (BMC) is blue/green and high BMC is pink; scale bar = 1mm. Graphs show relative bone mineral content (BMC, mean±SD) and femur length (median±2.5th and 97.5th percentiles) with WT reference range represented as grey violin plots with individual values shown as white dots (n=320). Individual values, together with the mean or median, from Pls3−/− (orange n=2) are shown. Significant P values after permutation testing are indicated. (b) Caudal vertebrae from P112 WT and Pls3−/− mice; scale bar = 1mm. Graphs show BMC (mean±SD), and vertebral length (mean±SD) with WT reference range represented as grey violin plots with individual values shown as white dots (n=320). Individual values and mean from Pls3−/− (orange n=2) mice are shown. (c) Micro-CT images of distal femur trabecular bone from P112 WT and Pls3−/− mice; scale bar = 100μm. Graphs show bone volume as a proportion of tissue volume (BV/TV, mean±SD), trabecular number (Tb.N, mean±SD), trabecular thickness (Tb.Th, mean±SD), and trabecular separation (Tb.Sp, median±2.5th and 97.5th percentiles). Significant P values after permutation testing are indicated. (d) Micro-CT images of femur mid-shaft cortical bone from P112 WT and Pls3−/− mice; scale bar = 100μm. Graphs show cortical thickness (Ct.Th, mean±SD), internal diameter (Int.Di, mean±SD), and bone mineral density (Ct.BMD, median±2.5th and 97.5th percentiles). Significant P values after permutation testing are indicated. (e) Representative load displacement curves from three-point bend testing of femurs from P112 WT (blue) and Pls3−/− (orange) mice. Graphs show yield load (mean±SD), maximum load (mean±SD), fracture load (median±2.5th and 97.5th percentiles), and stiffness (mean±SD). Significant P values after permutation testing are indicated. (f) Representative load displacement curves from compression testing of caudal vertebrae from P112 WT (bl [file media-17.pdf]

Supplementary Fig. 8. Skeletal vascular phenotyping of male *Pls3*<sup>y/-</sup> mice

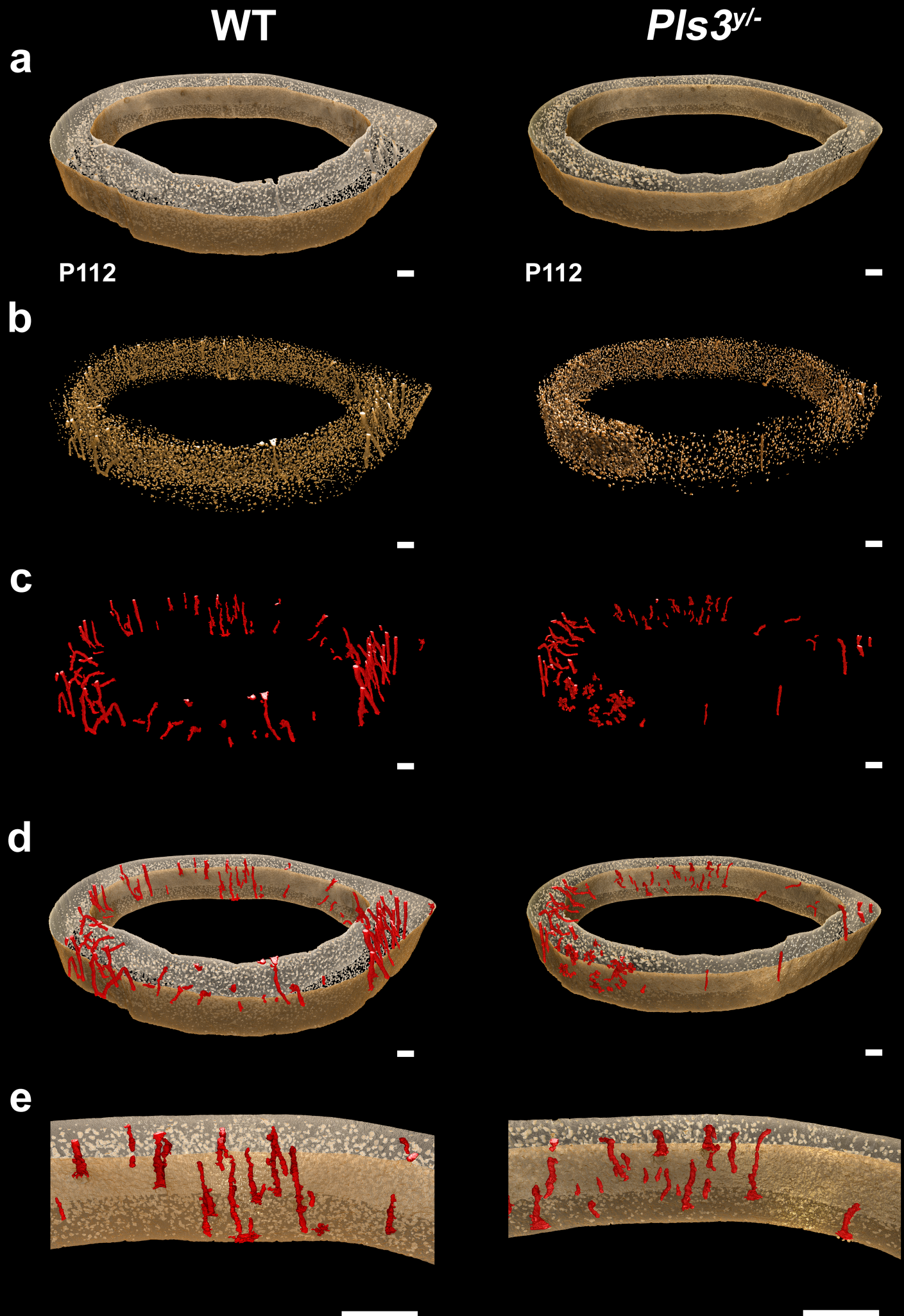

Supplement: Supplement 18 — (a) Micro-CT images (1.0 μm3 voxel resolution) of mid-femur cortical bone (250 μm long ROI) from P112 (WT n=6; Pls3y/− n=6) mice; scale bar = 100μm. (b) Osteocyte lacunae and cortical vascular canals, within the cortical bone ROI, with a volume greater than 100 μm3 identified using BoneJ Particle Analyser. (c) Cortical vascular canals (Red), within the cortical bone ROI, with a volume greater than 2000 μm3 identified using BoneJ Particle Analyser. (e) Overlay of mid-femur cortical bone ROI and vascular canals (f) Higher power image of cortical bone and vascular canals. [file media-18.pdf]
