# Supplementary material for: Multiscale analysis and functional validation of the cellular and genetic determinants of skeletal disease
Source: bioRxiv. 2026 Jun 1:2024.12.16.628792. Preprint. [Version 2] doi: 10.1101/2024.12.16.628792 (PMC13251937; doi:10.1101/2024.12.16.628792)

Extended Data Fig. 5. Gene programs of non-haematopoietic cell sub-clusters are enriched with monogenic skeletal disorder genes

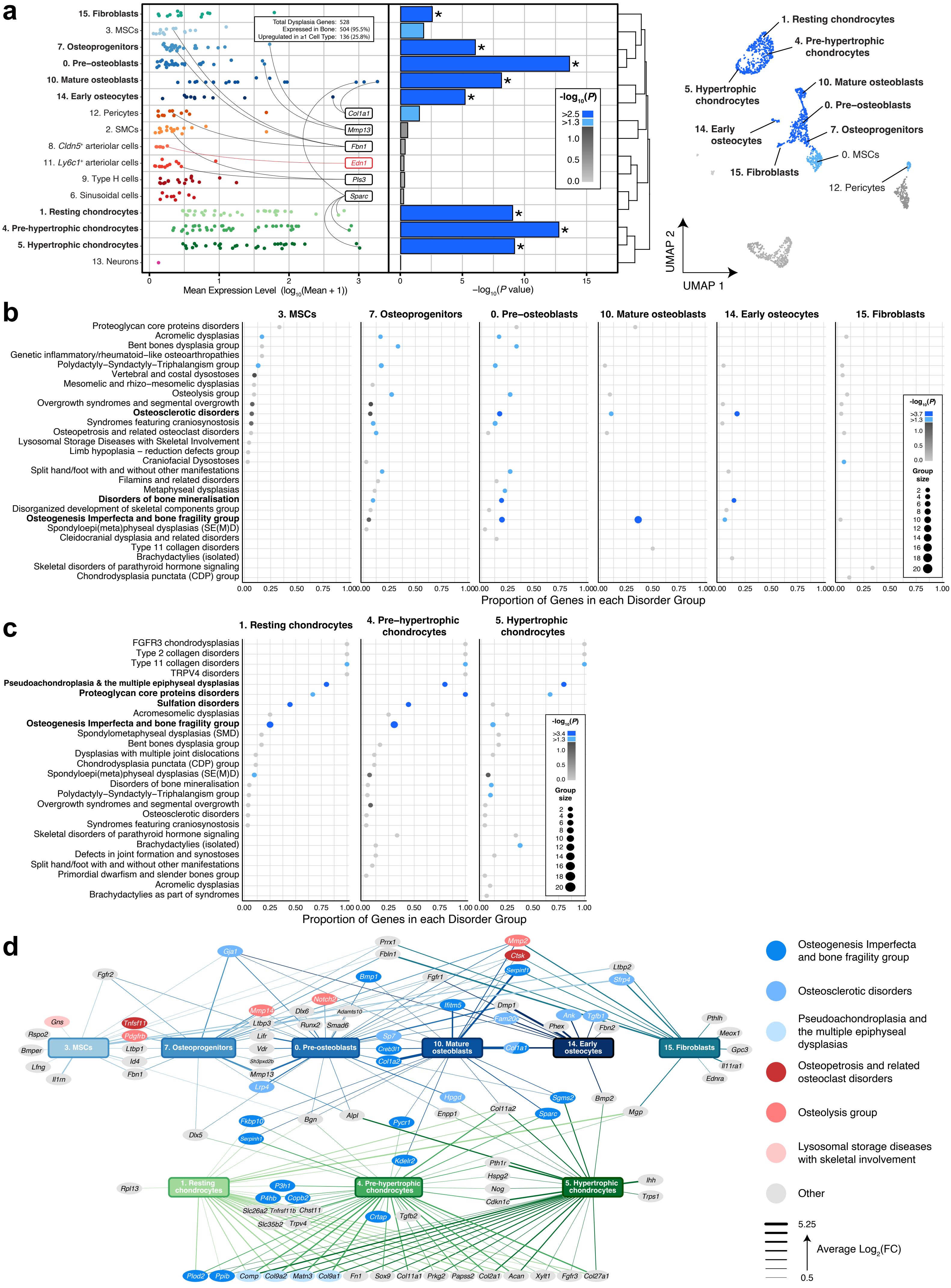

Supplement: Supplement 5 — (a) Dotplot showing the mean expression level (log10 mean+1) of causative genes of rare monogenic skeletal disorders for individual non-haematopoietic sub-cluster gene programs. Boxes identify exemplar causative genes present in a cell type gene program. Red text denotes a gene found only in a single gene program and not shared with other gene programs. Scale bar in bar plot and UMAP plot indicates the P value. Light blue bars in bar plot and light blue dots in UMAP correspond to observations that have nominal evidence of enrichment: P value of <0.05 [−log10(P value) of >1.3]. Dark blue bars and asterisks in bar plot and dark blue dots in UMAP correspond to observations that have robust evidence of enrichment and meet the Bonferroni-corrected significance threshold: P value of < 3.1 × 10^−3^ [−log10(P value) > 2.5]. Number and proportion of causative genes within rare disorder groups found in gene programs for osteoblast (b) and chondrocyte (c) sub-clusters. 27 and 26 disorder groups are identified for osteoblast and chondrocyte sub-clusters respectively, with those enriched with disorder-causing genes in at least one of the sub-clusters indicated in bold. Size of the circles represent the number of genes in each disorder group present within the gene program. Scale bar indicates the P value of enrichment, as determined by hypergeometric tests of over-representation. Light blue dots indicate nominal evidence of enrichment: P value of <0.05 [−log10(P value) of >1.3]. Dark blue dots denote robust evidence of enrichment with Bonferroni-corrected threshold of 2 × 10^−4^ (b) and 4.1 × 10^−4^ (c) [−log10(P value) of >3.7 (b) or > 3.4 (c)]. (d) Network plot showing unique and shared causative genes identified in the gene programs of osteoblast lineage and chondrocyte sub-clusters. Genes are colour coded based on disorder group and the magnitude of expression log2(FC) is indicated by the thickness of the connecting lines. [file media-5.pdf]
