# Supplementary material for: Multiscale analysis and functional validation of the cellular and genetic determinants of skeletal disease
Source: bioRxiv. 2026 Jun 1:2024.12.16.628792. Preprint. [Version 2] doi: 10.1101/2024.12.16.628792 (PMC13251937; doi:10.1101/2024.12.16.628792)

# Extended Data Fig. 7. Gene programs of non-haematopoietic cell sub-clusters are enriched with eBMD-associated genes

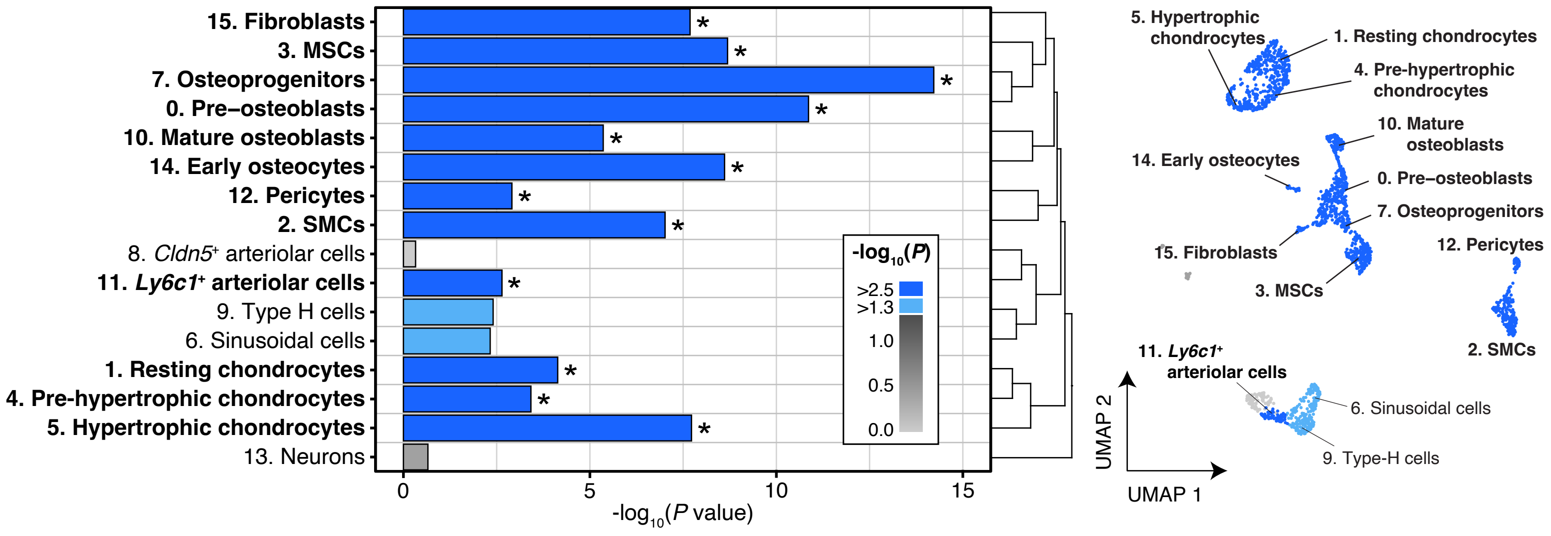

Supplement: Supplement 7 — Bar plot and UMAP plot showing enrichment of eBMD-associated genes in gene programs for different cell types identified in scRNA-seq. Scale bar in bar plot and UMAP plot indicates the P value. Light blue bars in bar plot and light blue dots in UMAP correspond to observations that have nominal evidence of enrichment: P value of <0.05 [−log10(P value) of >1.3]. Dark blue bars and asterisks in bar plot and dark blue dots in UMAP correspond to observations that have robust evidence of enrichment and meet the Bonferroni-corrected significance threshold: P value of < 3 ×10^−3^ [−log10(P value) > 2.5]. [file media-7.pdf]
