# Supplementary material for: Multiscale analysis and functional validation of the cellular and genetic determinants of skeletal disease
Source: bioRxiv. 2026 Jun 1:2024.12.16.628792. Preprint. [Version 2] doi: 10.1101/2024.12.16.628792 (PMC13251937; doi:10.1101/2024.12.16.628792)

Extended Data Fig. 8. Gene programs of non-haematopoietic cells and osteoclasts are enriched with genes that cause abnormal bone structure when mutated in mice

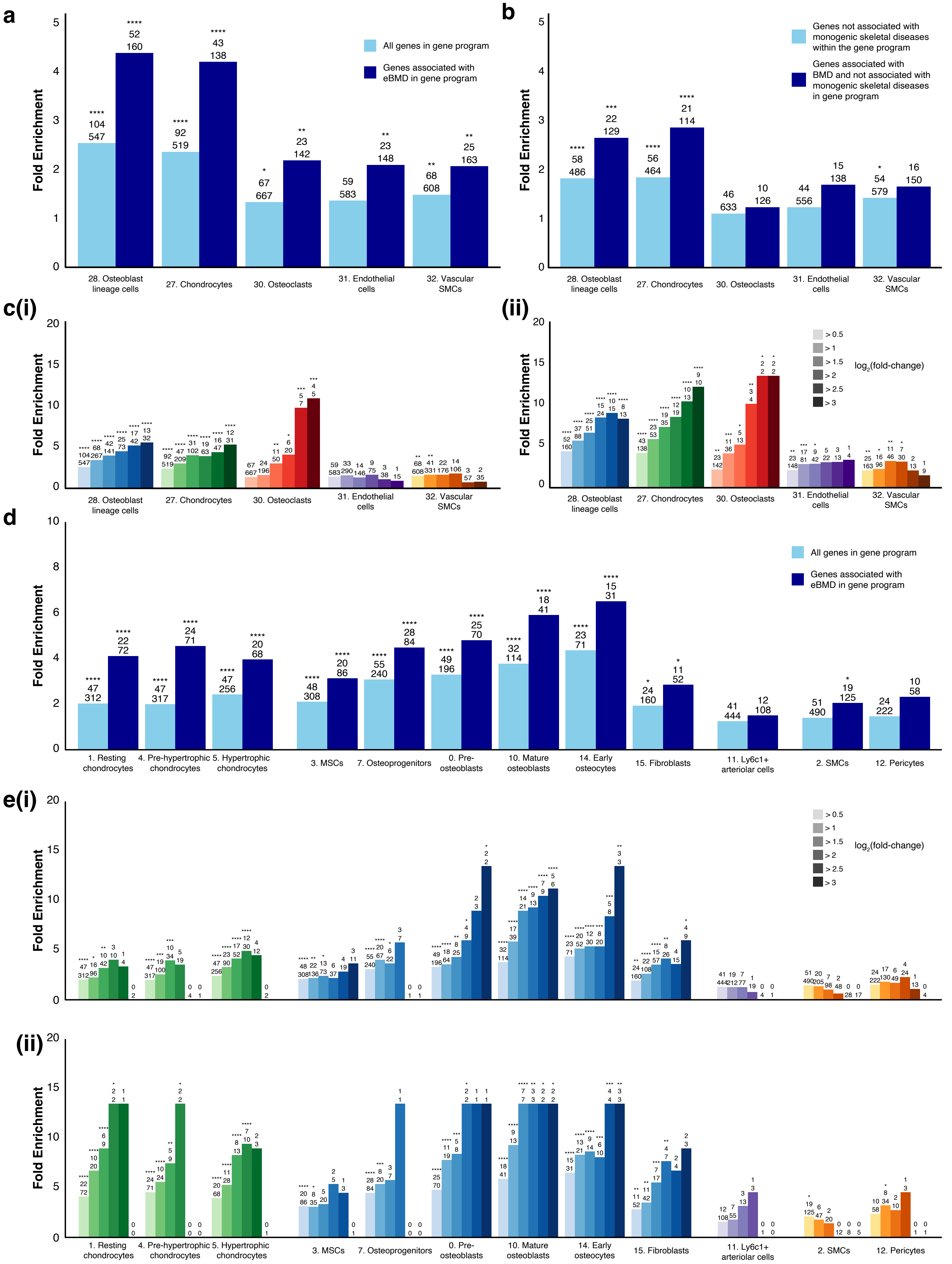

Supplement: Supplement 8 — (a) Enrichment of gene programs for genes that cause abnormal bone structure when mutated in mice in the MGI database. Light blue bars represent all genes in each gene program and dark blue bars represent genes in each gene program that are associated with eBMD in the MAGMA analysis. Numbers illustrate numbers of genes with abnormal bone structure phenotypes that are present in the gene program (top) and the total number of genes in each program (bottom). Enrichment was determined by hypergeometric over-representation testing. Bonferroni-corrected significance: **** P<0.0001, *** P<0.001, ** P<0.01, * P<0.05. (b) Enrichment of gene programs, excluding genes that are known to cause monogenic skeletal disorders, for genes that cause abnormal bone structure when deleted in mice in the MGI database. Light blue bars represent all genes in each gene program excluding causative genes for monogenic skeletal disorder; dark blue bars represent genes in each gene program that are not causative for monogenic skeletal disorders and are associated with eBMD in the MAGMA analysis. Numbers illustrate numbers of genes with abnormal bone structure phenotypes (top) and the total number of genes in each group (bottom). Enrichment was determined by hypergeometric over-representation testing. (c) Enrichment for genes that cause abnormal BMD when deleted in mice in the gene programs for non-haematopoietic cell types and osteoclasts, stratified by magnitude of fold-change in differential expression. Panel (i) includes all genes in the gene programs and panel (ii) the genes that are associated with eBMD in the gene programs. Genes within gene programs are allocated to 6 nested groups based on the magnitude of fold-change which increases from left to right. Numbers illustrate numbers of genes with abnormal bone structure phenotypes (top) and the total number of genes allocated to each group (bottom). Enrichment was determined by hypergeometric over-representation testing. Bonferroni-correcte [file media-8.pdf]
