# Supplementary material for: Multiscale analysis and functional validation of the cellular and genetic determinants of skeletal disease
Source: bioRxiv. 2026 Jun 1:2024.12.16.628792. Preprint. [Version 2] doi: 10.1101/2024.12.16.628792 (PMC13251937; doi:10.1101/2024.12.16.628792)

Supplementary Fig. 1. Validation of cell populations enriched in the endosteal compartment of mouse bone.

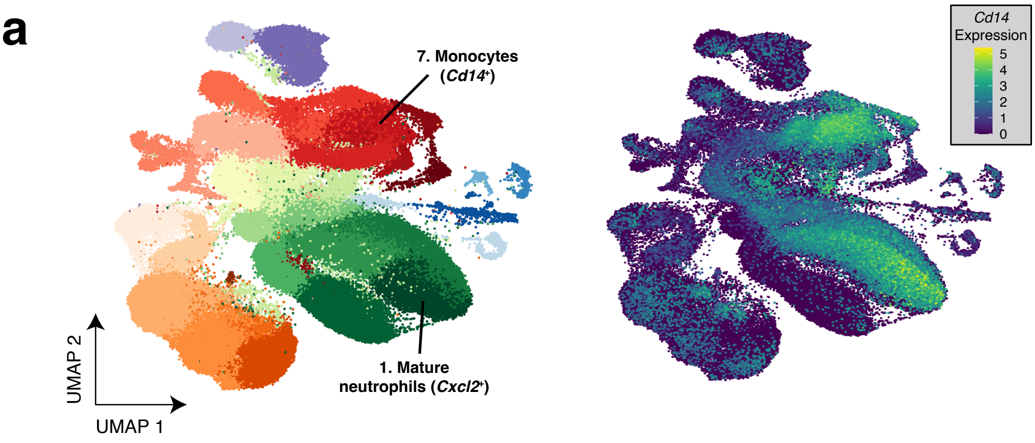

b

Cluster	Gene	Average Log_2FC	Adjusted p Value
7. Monocytes (*Cd14*+)	*Ctss*	2.76	0
	Ms4a6c	2.75	0
	Ms4a4c	2.64	0
	S100a4	2.63	0
	Ccr2	2.53	0
	Ccl9	2.53	0
	Apoe	2.40	0
	Ctsc	2.30	0
	Cd14	2.28	0
1. Mature neutrophils (*Cxcl2*+)	*Il1b*	3.39	0
	Dusp1	2.71	0
	Hcar2	2.58	0
	Ccl6	2.40	0
	Cebpb	2.40	0
	Egr1	2.28	0
	Slc7a11	2.27	0
	...	...	...
	Cd14	1.18	0

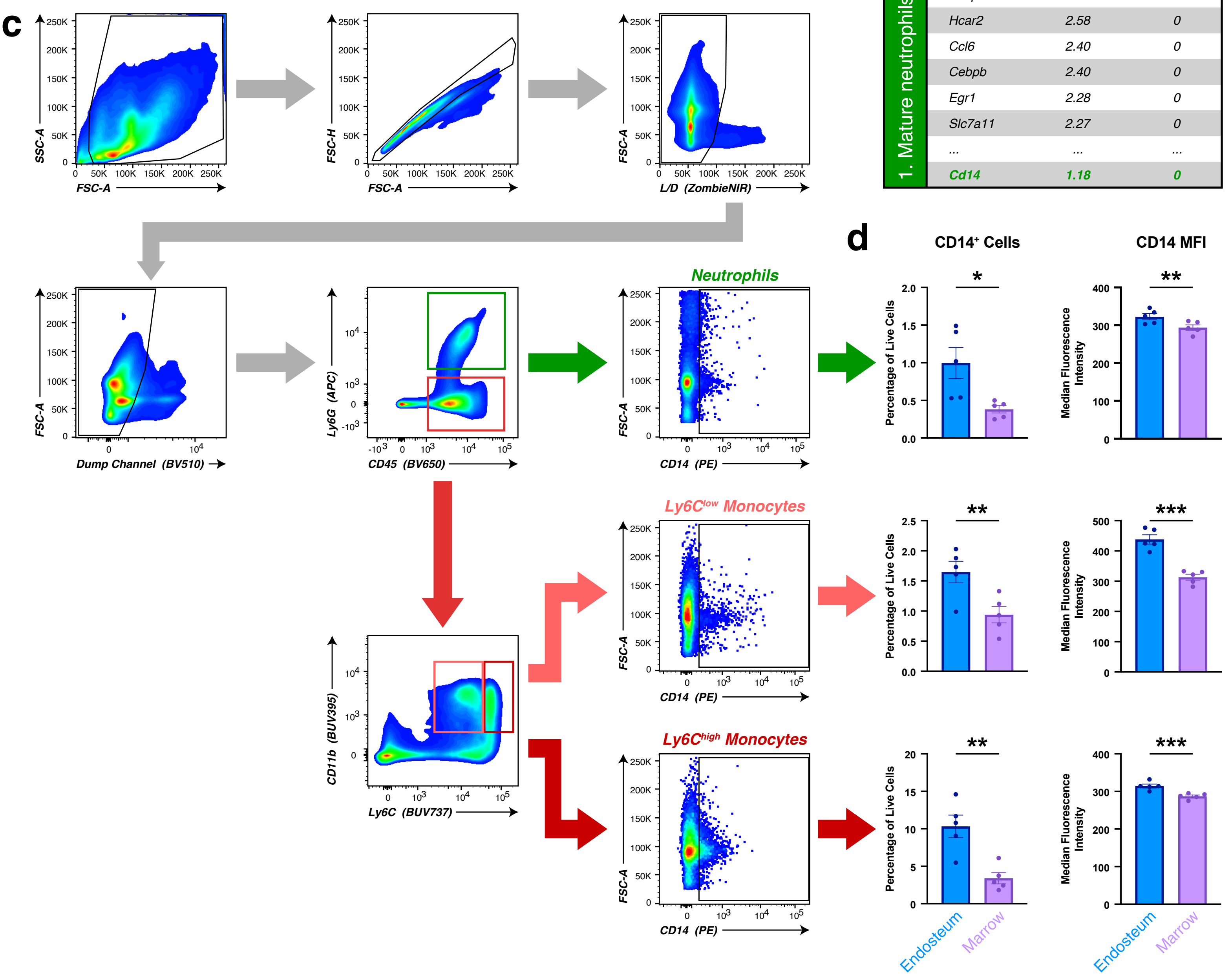

Supplement: Supplement 11 — (a) UMAP plots displaying expression of Cd14 within the mouse scRNA-Seq dataset. Clusters “7. Monocytes (Cd14+)” and “1. Mature neutrophils (Cxcl2+)” are indicated. (b) Top marker genes for clusters “7. Monocytes (Cd14+)” and “1. Mature neutrophils (Cxcl2+)”. Cd14 is highlighted as a strong marker for both clusters. (c) Gating strategy used to identify neutrophils and monocyte populations in mouse bone marrow and endosteal samples and to determine CD14 expression within these populations. (d) Quantification of CD14 expression in selected populations. Left bar plots show the proportion of cells with detectable CD14 expression. Right bar plots show median fluorescence intensity of CD14 within selected populations. Mean ± SEM are shown; Students’ t-test; *** P<0.001, ** P<0.01, * P<0.05. [file media-11.pdf]
