# Supplementary material for: Multiscale analysis and functional validation of the cellular and genetic determinants of skeletal disease
Source: bioRxiv. 2026 Jun 1:2024.12.16.628792. Preprint. [Version 2] doi: 10.1101/2024.12.16.628792 (PMC13251937; doi:10.1101/2024.12.16.628792)

# Supplementary Fig. 2. Distribution of non-haematopoietic cell sub-clusters in diaphysis and metaphysis

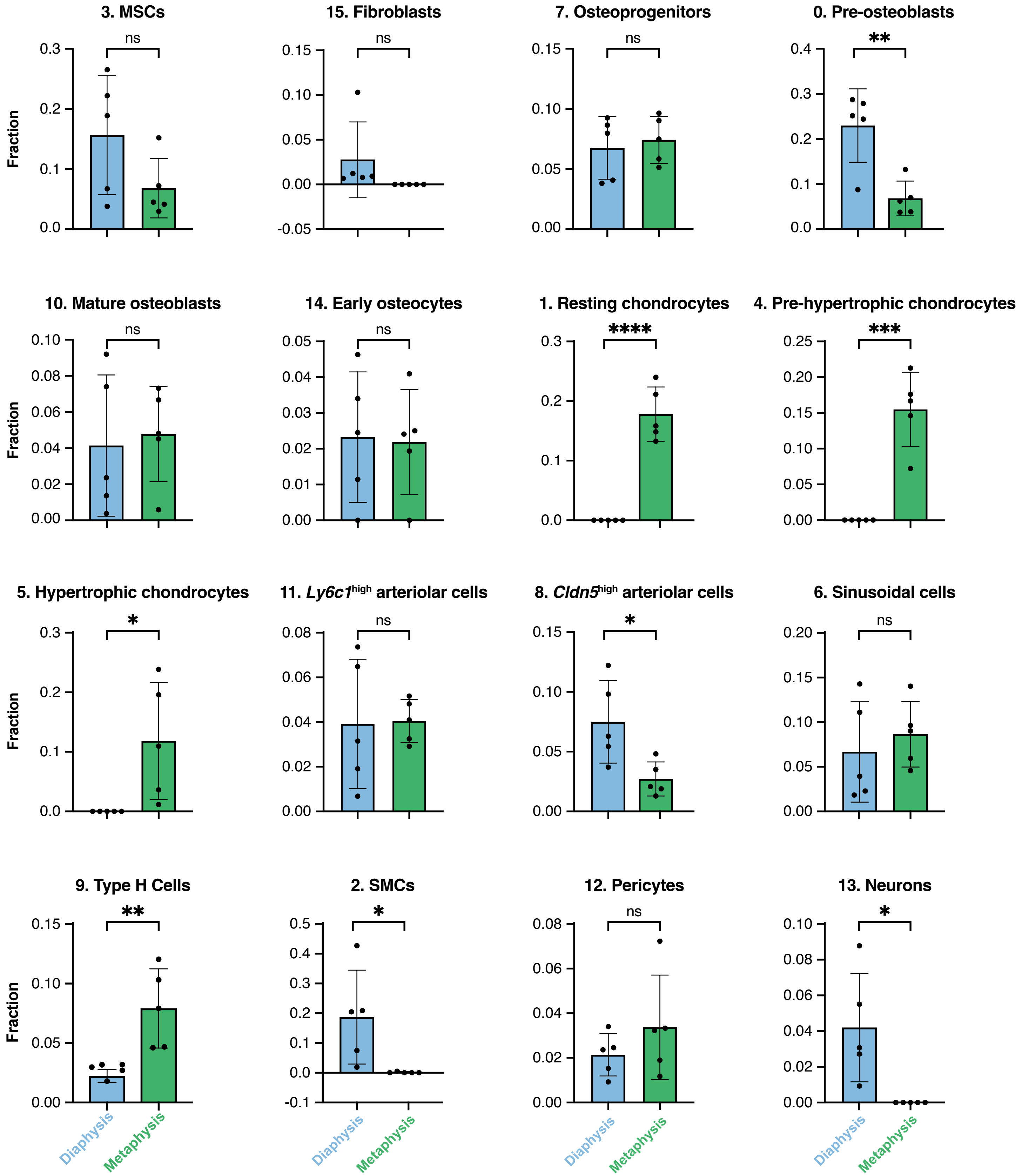

Supplement: Supplement 12 — Bar plots showing the fraction of all non-haematopoietic sub-clusters in diaphysis and metaphysis. Mean ± SEM are shown; Students’ t-test; **** P<0.0001, *** P<0.001, ** P<0.01, * P<0.05. [file media-12.pdf]
