# Supplementary material for: Multiscale analysis and functional validation of the cellular and genetic determinants of skeletal disease
Source: bioRxiv. 2026 Jun 1:2024.12.16.628792. Preprint. [Version 2] doi: 10.1101/2024.12.16.628792 (PMC13251937; doi:10.1101/2024.12.16.628792)

Supplementary Fig. 3. Genes associated with eBMD, but not pulse rate, are enriched with monogenic skeletal disorder genes

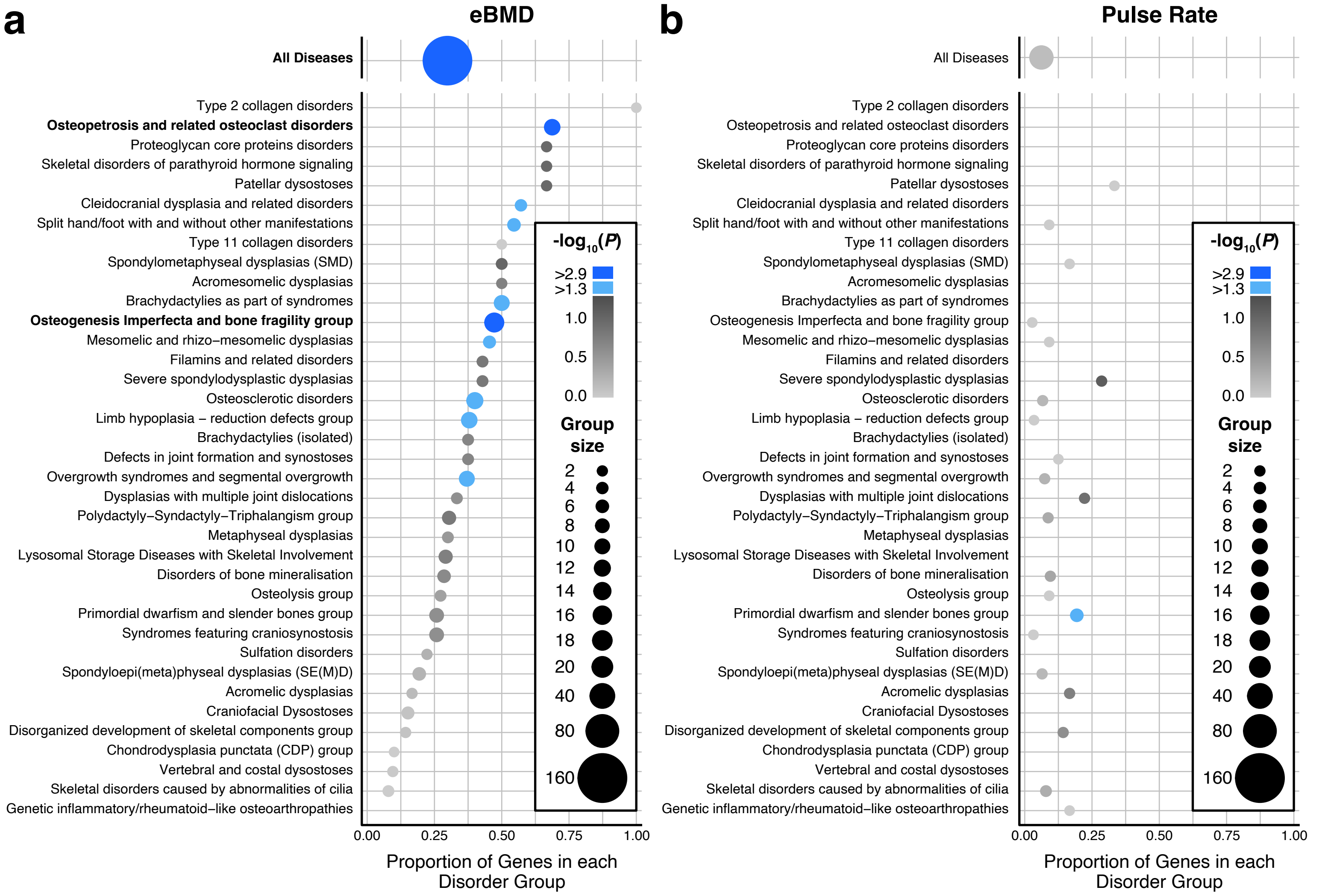

Supplement: Supplement 13 — Bubble plot showing the number, and proportion of monogenic skeletal disorder genes from each disorder group that are present in the set of protein-coding genes associated with (a) eBMD and (b) pulse rate. Size of the circles represent the number of genes in each disorder group present within the gene list. Scale bar indicates the P value of enrichment, as determined by hypergeometric over-representation testing. Light blue dots indicate nominal evidence of enrichment: P value of <0.05 [-log10(P value) of >1.3]. Dark blue dots denote robust evidence of enrichment with Bonferroni-corrected threshold of 1.2 × 10^−3^ [-log10(P value) of >2.9]. [file media-13.pdf]
