# Supplementary material for: Multiscale analysis and functional validation of the cellular and genetic determinants of skeletal disease
Source: bioRxiv. 2026 Jun 1:2024.12.16.628792. Preprint. [Version 2] doi: 10.1101/2024.12.16.628792 (PMC13251937; doi:10.1101/2024.12.16.628792)

Supplementary Fig. 4. Conditional GSA analysis to determine whether enrichment was confounded by shared sets of genes

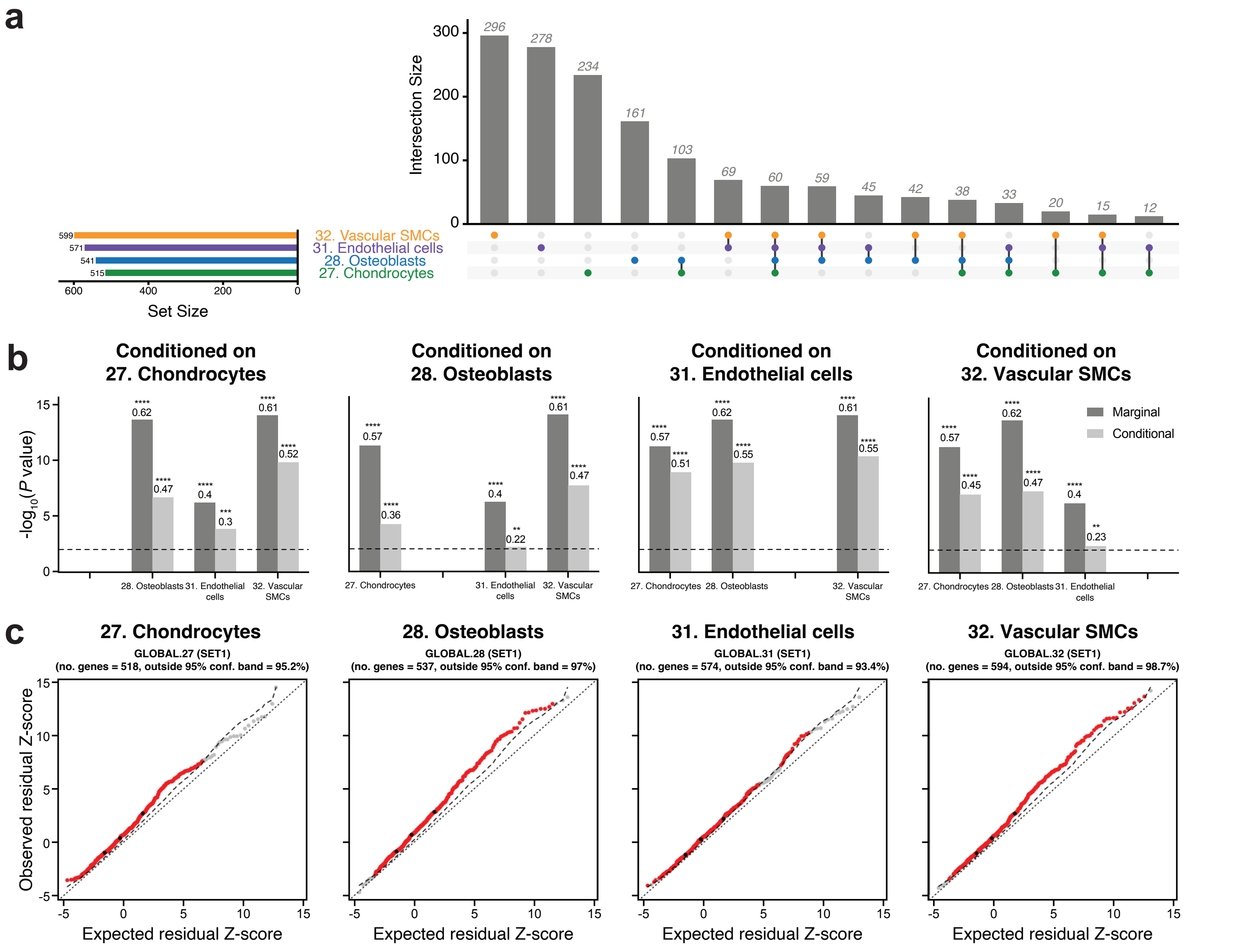

Supplement: Supplement 14 — (a) UpSet plot showing the number of restricted and shared genes associated with eBMD in non-haematopoietic cell types. (b) Histograms showing a pairwise conditional GSA analysis used to determine whether enrichment was confounded by shared sets of genes. Each histogram quantifies the strength of evidence of enrichment. Dark grey bars correspond to strength of evidence of enrichment in marginal (original) analyses and light grey bars correspond to the strength of evidence of enrichment after adjusting for the effect of genes that are shared between two cell types (conditional analyses). Numbers above each bar correspond to the GSA point estimate (i.e. β). **** P<0.001, *** P<0.005** P<0.01, * P<0.05. Dotted line corresponds to the threshold of statistical significance (P < 0.05). (c) Post-hoc permutation analyses showing QQ-plots of Z-scores of genes in each gene program of different cell clusters. Plots show residualised Z-scores from the null model for each gene program, with the expected values based on the quantiles across all genes in the data. The black points denote the 25th, 50th and 75th percentile. The dashed black line represents the one-sided (upper) 95% confidence band. Genes are coloured red if they exceed the confidence band, and grey if they do not. The proportion of genes in each program exceeding the confidence band is indicated on each plot. [file media-14.pdf]
