# Supplementary material for: Multiscale analysis and functional validation of the cellular and genetic determinants of skeletal disease
Source: bioRxiv. 2026 Jun 1:2024.12.16.628792. Preprint. [Version 2] doi: 10.1101/2024.12.16.628792 (PMC13251937; doi:10.1101/2024.12.16.628792)

Supplementary Note 3. Overview of the FACS strategy to collect live non-erythroid cells from mouse and human bones

a **Mouse**

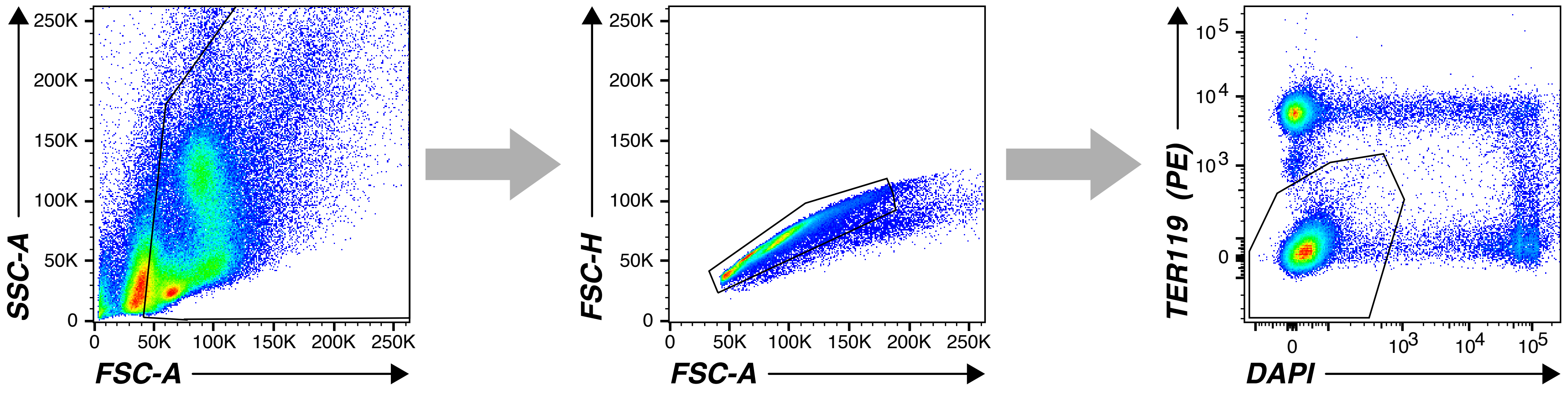

b **Human**

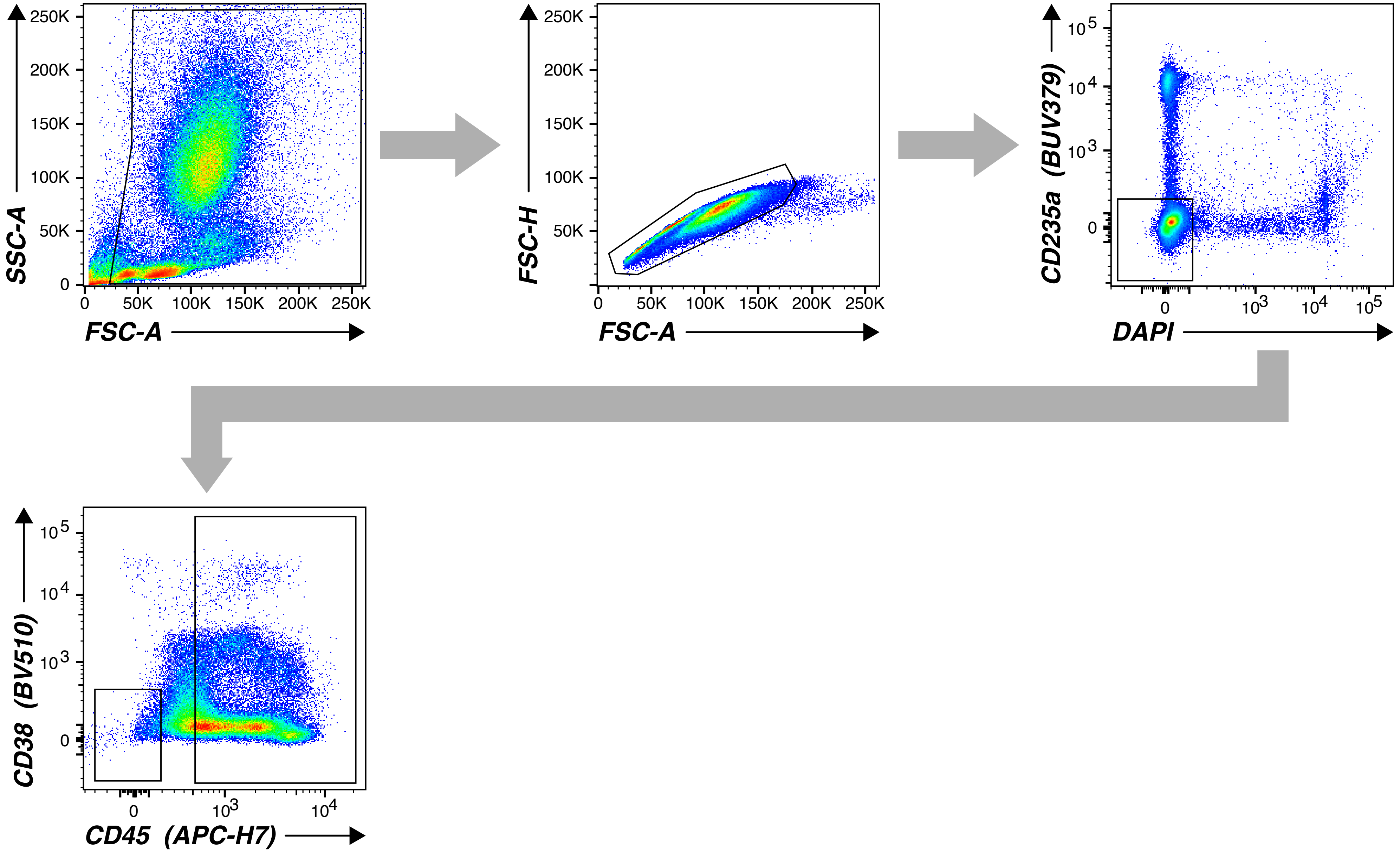

Supplement: Supplement 30 [file media-30.pdf]
